# Gut microbiota signatures across BMI categories in adults and combined diagnostic value with clinical indicators for early obesity risk stratification

**DOI:** 10.3389/fmicb.2025.1734612

**Published:** 2026-01-16

**Authors:** Xunuo Chen, Yonge Wang, Yongling Lv, Huawei Wang, Yanying Yang, Jun Tang, Hexiao Shen, Zhe Dai

**Affiliations:** 1 Department of Endocrinology, Zhongnan Hospital of Wuhan University, Wuhan University, Wuhan, Hubei, China; 2 School of Life Science, Hubei University, Wuhan, Hubei, China; 3 State Key Laboratory of Metabolism and Regulation in Complex Organisms, Wuhan University, Wuhan, Hubei, China; 4 Department of Clinical Nutrition, Zhongnan Hospital of Wuhan University, Wuhan University, Wuhan, Hubei, China

**Keywords:** clinical metabolic indicators, gut microbiota, microbial biomarkers, obesity, overweight

## Abstract

**Background:**

The global prevalence of obesity continues to rise, with gut microbiota implicated as a key environmental factor in its development. However, microbial heterogeneity across BMI categories and its potential as an early diagnostic marker remain poorly understood. Here, we systematically compared the gut microbiota of adults with normal weight, overweight, and obesity, and evaluated the diagnostic utility of integrated microbial and clinical indicators.

**Methods:**

In this study, a cohort of 214 participants was recruited and categorized into three groups based on BMI: normal weight (Nor, *n* = 85), overweight (Ow, *n* = 91), and obesity (Ob, *n* = 38). The gut microbiota was analyzed using 16S rRNA sequencing, which facilitated the assessment of *α*- and *β*-diversity, the identification of differentially abundant bacterial genera, and the evaluation of Spearman correlations with clinical indicators. Additionally, a diagnostic model was developed utilizing random forest, Least Absolute Shrinkage and Selection Operator (LASSO) regression and receiver operating characteristic (ROC) curve analysis.

**Results:**

Clinical data showed no significant age differences among groups (*p* > 0.05), but the Ob group had markedly higher weight, BMI, total cholesterol, triglycerides, and uric acid levels (*p* < 0.001). Microbial analysis revealed reduced alpha diversity (Shannon index) in the Ob group and distinct microbial community structures among groups (PCoA, *p* < 0.001). LEfSe analysis showed enrichment of *Escherichia-Shigella*, *Lactobacillus*, and *Parabacteroides* in the Ob group, while *Faecalibacterium*, *Enterobacter*, and *Agathobacter* were predominant in the Nor group. The Ow group exhibited intermediate or specific enrichment, particularly in *Ruminococcus gnavus group*, *Morganella*, and *Clostridium innocuum group*. Correlation analysis indicated that bacteria abundant in the Nor group negatively correlated with BMI, TG, LDL-C, and positively with HDL-C, whereas those enriched in the Ow and Ob groups showed opposite trends. Four genera differentiated Ob from Ow individuals (AUC = 0.684), eight distinguished Ob from Nor (AUC = 0.787), and five separated Ow from Nor (AUC = 0.721). Integrating microbial and clinical data notably improved classification, with AUCs increasing to 0.908, 0.969, and 0.995, respectively.

**Conclusion:**

Intestinal microecological profiles vary significantly across BMI categories, and a model combining microbiota with metabolic markers shows strong potential for early obesity detection.

## 1 Introduction

Obesity is a widespread metabolic disorder characterized by chronic and complex traits. Its etiology is multifactorial, encompassing genetic, environmental, and behavioral influences, and its prevalence is on the rise globally (Janić et al., 2025). This condition serves as a significant risk factor for various health issues, including cardiovascular disease, type 2 diabetes, non-alcoholic fatty liver disease, and certain cancers (Alexander et al., 2019; Wang et al., 2024; Yu et al., 2024; Leitzmann et al., 2025; Mo et al., 2025). The World Obesity Federation (2025) report projects that the number of adults with elevated body mass index (BMI) worldwide will increase to 2.9 billion by 2030. Similarly, China is experiencing a parallel trend, with the rates of obesity and overweight individuals expected to reach 70.5% by 2030 (World Obesity Federation, 2025). Furthermore, there is a concerning annual increase in the number of overweight adolescents and children, with a noticeable trend towards younger populations becoming obese [NCD Risk Factor Collaboration (NCD-RisC), 2024]. This situation poses a substantial burden on global public health systems. Consequently, it is of paramount clinical and social importance to gain a comprehensive understanding of the pathogenesis of obesity and to identify new targets for early intervention.

In recent years, advancements in microbiome research and high-throughput sequencing technologies have positioned gut microbiota as a significant factor influencing host metabolism and immune function, thereby emerging as a prominent subject of investigation in the field of metabolic disease research. The gut microbiota is essential for modulating energy intake and expenditure, regulating metabolic processes and inflammatory responses, and preserving the integrity of the intestinal barrier (Carmody and Bisanz, 2023; Wu et al., 2024a). Research indicates that germ-free mice subjected to fecal microbiota transplantation for the purpose of addressing obesity exhibited a marked increase in body weight and associated obesity symptoms, thereby implying a potential causal relationship between gut microbiota and obesity (Borrello et al., 2022; Yarahmadi et al., 2024). Furthermore, investigations have revealed that individuals with obesity often present with dysbiosis in their gut microbiota, accompanied by alterations in the abundance of critical bacterial genera. Recent extensive metagenomic analyses have identified *Megamonas* as a potential “obesogenic bacterium” among obese individuals in China, and its enrichment is significantly positively correlated with BMI and waist circumference (Wu et al., 2024b). Similarly, *Enterobacter cloacae* has been identified as a significant contributor to obesity. Research indicates that this bacterium constitutes approximately one-third of the total microbial population in the intestines of individuals with obesity. Furthermore, studies have demonstrated that *Enterobacter cloacae* is capable of inducing obesity and exacerbating inflammation in murine models (Fei and Zhao, 2013). Individuals with obesity frequently exhibit a reduction in the presence of anti-obesity bacteria, including species such as *Bacteroides thetaiotaomicron* and *Faecalibacterium prausnitzii* (Liu et al., 2017; Song et al., 2023). And, research indicates that the abundance of *Faecalibacterium prausnitzii* is significantly and negatively correlated with both weight loss and the reduction of fat mass (Kahleova et al., 2020); the prevalence of *Bacteroides thetaiotaomicron* demonstrates a negative correlation with serum glutamate levels, which are associated with fat accumulation and weight gain (Liu et al., 2017). Furthermore, an increasing body of research has demonstrated that the intestinal microbiota engage in interactions with the host through the production of metabolites (Nicholson et al., 2012). Microorganisms are capable of generating essential metabolites, including short-chain fatty acids (SCFAs) and bile acid derivatives, through the fermentation of dietary fiber (Delzenne et al., 2025). SCFAs, particularly propionic acid and butyric acid, play a significant role in lipid metabolism by modulating hypothalamic activity via the blood–brain barrier, thereby decreasing food intake and inhibiting lipogenesis (Mukhopadhya and Louis, 2025). Likewise, the synthesis of secondary bile acids has been associated with enhanced energy expenditure and a reduction in fat accumulation (Ticho et al., 2019). In general, current research has established that individuals with obesity frequently exhibit specific traits, including dysbiosis in microbial diversity, a diminished presence of butyrate-producing bacteria (notably *Faecalibacterium*) and bacteria responsible for producing secondary bile acids, alongside an elevated abundance of pro-inflammatory bacteria. This phenomenon is referred to as the “obesity-type microecological imbalance” (Breton et al., 2022)^.^

Existing research has established a significant relationship between obesity and intestinal microecology; however, there is considerable variability in the microecological profiles among individuals with obesity. Initial investigations highlighted an enrichment of Firmicutes in obese populations, accompanied by a reduction in Bacteroidetes, leading to the conclusion that an elevated Firmicutes/Bacteroidetes ratio is a characteristic feature of obesity (Breton et al., 2022). Recent research has introduced alternative viewpoints, with certain findings indicating an elevation in Bacteroidetes among individuals with obesity, or proposing that there is no significant variation in the Firmicutes/Bacteroidetes ratio (Magne et al., 2020). Correspondingly, a prevailing agreement among various studies indicates that individuals with obesity tend to demonstrate a reduced diversity of intestinal microbiota. However, research conducted by Japanese scholars has demonstrated that the diversity of intestinal microbiota in individuals with obesity is significantly higher than that found in individuals without obesity (Uema et al., 2022). These divergent findings highlight the variability in research results concerning intestinal microorganisms among various populations of individuals with obesity.

The alterations and composition of intestinal microbiota are influenced by a variety of factors, including demographic variations (such as ethnicity and geographic location), dietary patterns, lifestyle choices, sample size, and the methodologies employed in analysis (Brooks et al., 2018; Dwiyanto et al., 2021; Borrello et al., 2022; Andreu-Sánchez et al., 2025). More importantly, a study has demonstrated notable variations in the diversity and composition of gut microbiota among individuals with differing BMIs (Wang et al., 2025). Nonetheless, there remain significant gaps in the existing systematic research concerning the relationship between BMI and intestinal microbiota in China. Notably, studies that incorporate normal, overweight, and obese populations within a cohesive framework for comparative analysis are particularly limited. Furthermore, there is a deficiency in the investigation of microbial markers that could facilitate early intervention during the overweight phase. It is also important to acknowledge that BMI does not provide a direct measure of adipose tissue. Individuals with comparable BMI values may exhibit divergent health outcomes, indicating the necessity for additional metrics to assess the health implications associated with overweight and obesity (Sweatt et al., 2024). Consequently, to address these gaps, it is essential not only to undertake systematic research across BMI strata but also to explore the potential of gut microbiota, combined with routine metabolic markers, for stratifying metabolic health independent of BMI itself.

Our study was designed with two primary innovative aims: first, to systematically identify the unique and transitional gut microbial signatures of the overweight state, a critical yet understudied pre-obesity phase; and second, to develop and evaluate a predictive model that integrates these microbial markers with clinical metabolic indicators for obesity stratification, deliberately excluding BMI to test its intrinsic utility. By identifying core intestinal microorganisms associated with different BMI categories and clinical metabolic indicators independent of BMI, we developed a predictive model that integrates microbial markers significantly correlated with both BMI and clinical indicators. This study offers theoretical insights and identifies potential biomarkers for the early detection and targeted intervention of obesity, highlighting the variations in intestinal microorganisms among individuals with differing BMI classifications.

## 2 Methods and materials

### 2.1 Volunteer recruitment

This research received approval from the Ethics Committee of Zhongnan Hospital (Code: 2025054) and was carried out in strict compliance with the principles outlined in the Declaration of Helsinki. Participants classified as overweight or obesity were recruited from the Weight Loss and Metabolism Center at Zhongnan Hospital, while the normal control group was sourced from the Physical Examination Department. It is noteworthy that all participants had not engaged in any specific dietary interventions in the 12 months preceding their recruitment and maintained a varied diet to accurately reflect the intestinal flora composition associated with their typical dietary practices. Inclusion criteria: (1) participants aged between 18 and 60 years, irrespective of gender; (2) individuals must be in good health, without significant organic diseases or chronic infectious conditions; (3) participants must not have received antibiotics, probiotics, or medications that regulate intestinal function within the 3 months prior to sampling; (4) participants were categorized by BMI in accordance with the most recent guidelines for the diagnosis and treatment of obesity (Department of Medical Administration, National Health Commission of the People′s Republic of China, 2024): normal group (Nor): 18.5 ≤ BMI < 24.0 kg/m^2^; overweight group (Ow): 24.0 ≤ BMI < 28.0 kg/m^2^; Obesity group (Ob): BMI ≥ 28.0 kg/m^2^. Exclusion criteria: (1) individuals who are pregnant or currently breastfeeding; (2) participants who have received antibiotics, probiotics, or immunomodulators, or who have undergone significant surgical interventions within the last 3 months; (3) individuals with a documented history of gastrointestinal disorders, including inflammatory bowel disease and constipation, as well as metabolic disorders such as type 2 diabetes and hypertension, and major systemic diseases such as cancers, chronic liver disease, or renal disease; (4)individuals with a history of substance abuse, alcoholism, or psychiatric or neurological disorders; and (5) individuals exhibiting atypical dietary practices or imbalanced dietary patterns, such as strict vegetarians or those following extreme high-fat diets. The clinical characteristics of all participants are presented in Table 1.

**Table 1:** Comparison of baseline characteristics among different BMI groups.

Characteristics	Ob (*n* = 38)	Ow (*n* = 91)	Nor (*n* = 85)	*p*-value
Male/female	26/12	47/44	33/52	***–***
Age	38.05 ± 10.33	41.81 ± 10.54	42.51 ± 11.32	0.092
BMI	34.85 ± 5.49	25.49 ± 1.17	21.81 ± 1.57	<0.0001
WBC	6.44 ± 1.79	5.79 ± 1.52	5.49 ± 0.87	0.02
HGB	147.16 ± 15.71	142.35 ± 27.48	134.48 ± 12.84	0.00016
PLT	267.58 ± 64.89	236.19 ± 63.54	237.53 ± 53.94	0.02169
Alb	46.43 ± 2.89	44.05 ± 4.28	42.33 ± 3.35	<0.0001
TC	4.57 ± 1.26	4.96 ± 1.06	4.31 ± 0.62	0.00045
LDL-C	3.03 ± 0.79	3.15 ± 0.92	2.47 ± 0.47	<0.0001
HDL-C	1.19 ± 0.85	1.23 ± 0.38	1.71 ± 0.24	<0.0001
TG	2.56 ± 3.02	2.07 ± 2.15	1.19 ± 0.30	<0.0001
Cr	65.57 ± 14.43	64.93 ± 13.64	65.02 ± 14.13	0.858
Ua	473.23 ± 124.96	365.57 ± 98.08	310.47 ± 55.34	<0.0001
Urea	4.92 ± 1.54	4.70 ± 1.35	5.05 ± 1.32	0.15536
Glu-AC	7.64 ± 1.81	6.26 ± 1.37	5.19 ± 0.47	<0.0001
SBP	126.79 ± 12.52	121.11 ± 11.27	117.36 ± 5.78	0.00032
DBP	85.11 ± 10.06	78.65 ± 8.40	80.22 ± 5.68	0.000384

BMI: body mass index; WBC: white blood cell count; HGB: hemoglobin; PLT: platelet count; Alb: albumin; TC: total cholesterol; LDL-C: low-density lipoprotein cholesterol; HDL-C: high-density lipoprotein cholesterol; TG: triglycerides; Cr: creatinine; Ua: uric acid; Urea: urea nitrogen; Glu-AC: fasting blood glucose; SBP; Systolic blood pressure; DBP; diastolic blood pressure. The data were analyzed using the Kruskal–Wallis test, with *p* < 0.05 considered statistically significant.

### 2.2 Sample collection

Fecal samples were obtained utilizing a sterile stool collection kit. Participants were instructed to collect stool samples in their homes, following the provided guidelines. They utilized a sampling spoon to gather 1–2 g of stool, which was subsequently placed into a stool sampling tube. The samples were then promptly stored in the freezer of a domestic refrigerator at approximately −20 °C. Within 24 h, the samples were transported to the laboratory on dry ice and subsequently stored at −80 °C until analysis.

### 2.3 DNA extraction, PCR amplification and sequencing

Genomic DNA was isolated from stool samples utilizing the HiPure Stool DNA Mini Kit (Magen, Guangzhou, China), adhering closely to the protocols outlined by the manufacturer. Following the extraction process, the concentration of the DNA was quantified using the Qubit 4 Fluorometer (Thermo Fisher Scientific, United States) in conjunction with the Qubit dsDNA HS Assay Kit (Thermo Fisher Scientific, United States), which is designed to provide high sensitivity for the detection of low-concentration DNA. Afterwards the integrity of genomic DNA was assessed through 1% agarose gel electrophoresis. Following this, the V3-V4 hypervariable region of the bacterial 16S rRNA gene was amplified via polymerase chain reaction (PCR) utilizing universal primers (341F, 5′-CCTACGGGNGGCWGCAG-3′ and 805R, 5′-GACTACHVGGGTATCTAATCC-3′). The PCR was conducted using KAPA HiFi HotStart ReadyMix (Roche, Switzerland) and was executed on a Veriti 96-well gradient PCR instrument (Applied Biosystems, United States). The resulting amplified PCR products underwent library construction, were purified using AMPure XP magnetic beads (Beckman Coulter, United States), and were subsequently subjected to paired-end sequencing (2 × 250 bp) on the Illumina MiSeq PE250 platform.

### 2.4 Data analysis

Raw sequencing data were processed in QIIME2 (version 2021.2.0). Quality control was performed using the DADA2 plugin, which removed low-quality reads and chimeras and generated high-quality, non-redundant amplicon sequence variants (ASVs). After denoising, the minimum paired-end sequencing depth across all samples was approximately 50,000 reads. Taxonomic assignment was conducted against the SILVA database (version 138).

To ensure comparability across samples, all *α*- and *β*-diversity analyses were performed after rarefying samples to a depth of 20,000 reads. Alpha diversity was quantified using the Shannon index to evaluate within-sample microbial diversity. Beta diversity was assessed using the Bray–Curtis distance matrix, visualized by principal coordinate analysis (PCoA). Differences in community structure among groups were tested using ANOSIM and Adonis. To identify differentially abundant taxa across BMI categories, Linear Discriminant Analysis Effect Size (LEfSe) was applied.

Spearman correlation analysis was conducted to examine the relationships between microbial genera and clinical parameters, including LDL-C, TC, TG, and BMI. To further identify microbial or metabolic markers capable of distinguishing Nor, Ow, and Ob individuals, a random forest classifier was constructed at the genus level, followed by feature selection using LASSO regression. Receiver Operating Characteristic (ROC) analyses were performed to determine the predictive performance of each model.

To investigate whether metabolic and microbial markers could stratify obesity independently of BMI, BMI was excluded from the diagnostic model and used solely as a grouping variable. A multivariate model integrating microbial markers with metabolic indicators (TG, HDL-C, UA) was then developed, and its discriminatory capacity was evaluated using the area under the curve (AUC).

### 2.5 Statistical analysis

Statistical analyses were performed using R software (version 4.3.2). Group differences in clinical variables and alpha diversity indices were assessed using the Kruskal–Wallis test. Beta diversity differences were evaluated using ANOSIM and Adonis based on the Bray–Curtis distance matrix. For multiple testing in differential abundance and correlation analyses, *p*-values were adjusted using the Benjamini–Hochberg false discovery rate (FDR) method. Model performance from random forest, LASSO, and ROC analyses was quantified using the area under the curve (AUC), and using logistic regression analyses employing Firth’s penalized likelihood estimation were used to obtain bias-reduced, finite parameter estimates. Statistical significance was defined as *p* < 0.05 or FDR-adjusted *q* < 0.05.

## 3 Results

### 3.1 Baseline characteristics of participants

In this study, a total of 214 volunteers were recruited, comprising 38 individuals in the Ob group, 91 in the Ow group, and 85 in the Nor group (Table 1). The BMI values among the three groups exhibited statistically significant differences (*p* < 0.0001), while no significant age differences were observed (*p* = 0.092). The fasting blood glucose (Glu-AC) levels in the Ob group (7.64 ± 1.81 mmol/L) were significantly higher than those observed in the Ow group (6.26 ± 1.37 mmol/L) and the Nor group (5.19 ± 0.47 mmol/L). Additionally, lipid profiling indicated persistent abnormalities, with the Ob group demonstrating the highest levels of triglycerides (TG, 2.56 ± 0.82 mmol/L) and low-density lipoprotein cholesterol (LDL-C, 3.03 ± 0.79 mmol/L), as well as the lowest levels of high-density lipoprotein cholesterol (HDL-C, 1.19 ± 0.21 mmol/L) among all groups (*p* < 0.0001). The Ob group exhibited significantly elevated serum albumin (Alb, 46.43 ± 2.89 g/L) and uric acid (Ua, 473.23 ± 124.96 μmol/L) levels compared to Ow (Alb, 44.05 ± 4.28 g/L, Ua, 365.57 ± 98.08 μmol/L) and Nor groups (Alb, 42.33 ± 3.35 g/L, Ua, 310.47 ± 55.34 μmol/L) (*p* < 0.0001), confirming metabolic dysregulation in Ob. Notably, the Ob group demonstrated concurrent increases in hematological parameters (WBC: 6.44 ± 1.79 × 10^9^/L; HGB: 147.16 ± 15.71 g/L; PLT: 267.58 ± 64.89 × 10^9^/L) and blood pressure (SBP: 126.79 ± 12.52 mmHg; DBP: 85.11 ± 10.06 mmHg) relative to Ow (121.11 ± 11.27/78.65 ± 8.40 mmHg) and Nor (117.36 ± 5.78/80.22 ± 5.68 mmHg) groups. These findings suggest a triad of pathological manifestations in obesity: chronic low-grade inflammation, prothrombotic tendency, and elevated cardiovascular risk. No significant intergroup differences were observed in serum creatinine (Cr, *p* = 0.858) or urea nitrogen (urea, *p* = 0.155) levels among the Ob, Ow, and Nor groups, suggesting preserved renal function in Ob individuals. However, the Ob group exhibited a characteristic metabolic triad: systemic dysregulation (evidenced by elevated Glu-AC and lipid profiles), chronic low-grade inflammation (reflected by increased WBC count), and elevated cardiovascular risk (demonstrated by higher blood pressure). Notably, while the Ow group showed intermediate abnormalities in certain parameters compared to the Nor group, the degree of abnormality was substantially smaller than in the Ob group, suggesting Ow status as a critical window for preventive intervention.

### 3.2 Gut microbiota diversity

**Figure 1:**
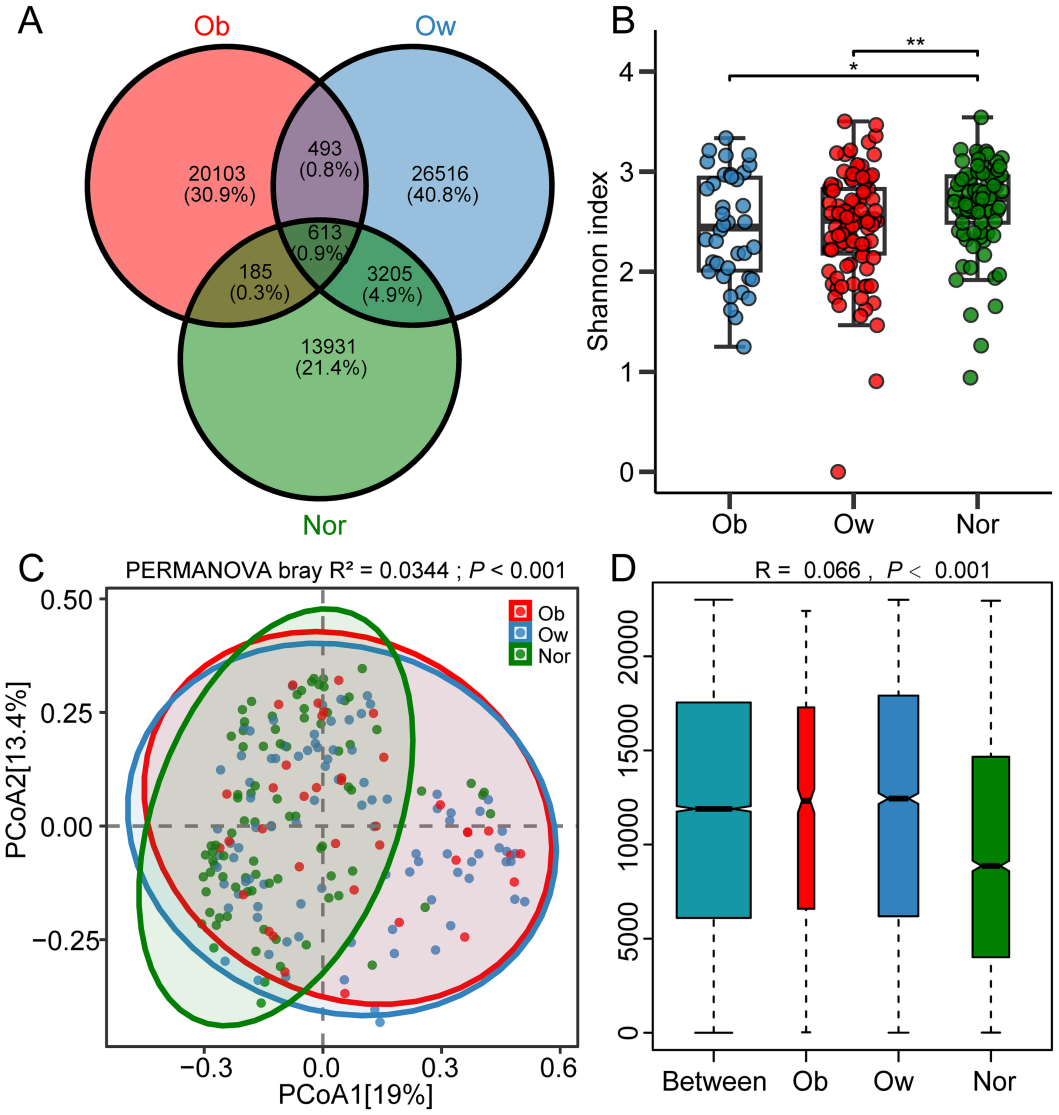
Gut microbiota characteristics across BMI groups. **(A)** Venn diagram illustrating the distribution of amplicon sequence variants (ASVs) among obesity, overweight, and normal-weight groups, with unique and shared ASV counts annotated. **(B)** Box plots of the Shannon diversity index (*α*-diversity) across the three groups. **(C)** Principal coordinate analysis (PCoA) based on Bray–Curtis dissimilarity depicting *β*-diversity differences among groups. **(D)** ANOSIM results showing microbial community segregation (*R* statistic and *p*-value indicated; Bray–Curtis distance used). Higher *R* values indicate stronger between-group separation. Ob, *n* = 38; Ow, *n* = 91; Nor, *n* = 85. Statistical significance was assessed using the Kruskal–Wallis test, followed by the Benjamini–Hochberg procedure. * p < 0.05; ** p < 0.01.

High-throughput sequencing of 214 fecal samples generated 13,027,572 high-quality 16S rRNA sequences, which were clustered into 97,141 ASVs. Venn analysis demonstrated distinct ASV distribution patterns among Ob, Ow, and Nor groups (Figure 1A), with the Ob group exhibiting the highest proportion of unique ASVs (28.7% vs. Ow 15.4% and Nor 9.8%). The Ob, Ow, and Nor groups contained 20,103 (30.9%), 26,516 (40.8%), and 13,931 (21.4%) unique ASVs, respectively, with only 613 ASVs (0.9%) shared among all three groups. Notably, the Nor and Ow groups shared 3,205 ASVs (4.9%), significantly more than the 185 ASVs (0.3%) shared between the Nor and Ob groups, demonstrating closer microbial community similarity between the Nor and Ow groups than between the Nor and Ob groups. The box plot of Shannon index demonstrated significant differences in *α*-diversity among Ob, Ow, and Nor groups (Figure 1B), with the Ob group exhibiting the lowest median value. PCoA based on Bray-Curtis distance revealed distinct clustering patterns, where both Ob and Ow groups showed significant separation from the Nor group along PCoA1 axis (Figure 1C, *p* < 0.001). ANOSIM analysis further confirmed the inter-group differences (Figure 1D, *R* = 0.066, *p* < 0.001), indicating statistically valid grouping with microbial community variations exceeding intra-group heterogeneity.

### 3.3 Taxonomic composition of the gut microbiota

**Figure 2:**
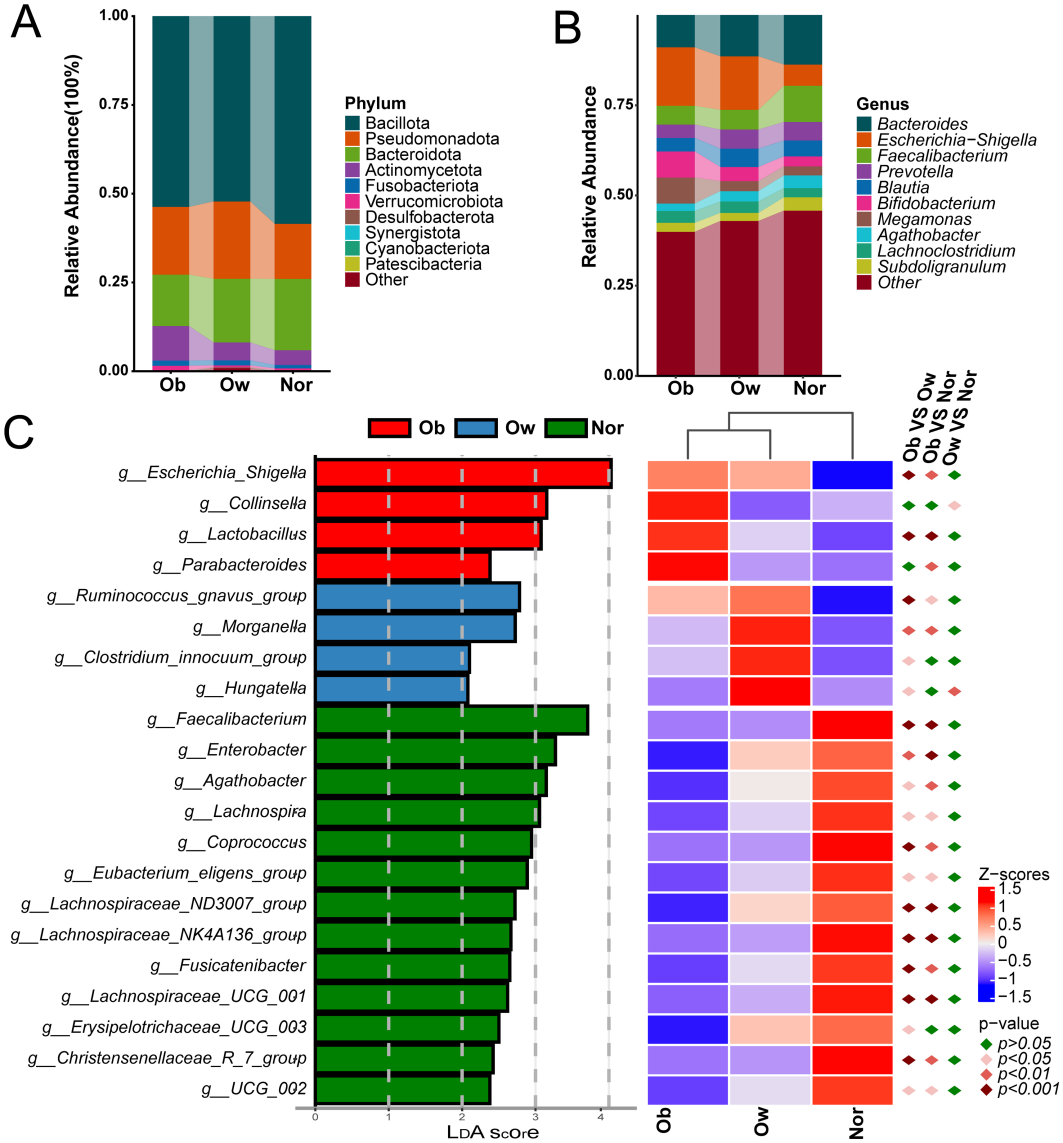
Differences analysis of gut microbial community structure in Ob, Ow, and Nor people. **(A)** Relative abundance histogram of the top 10 phyla in each BMI group. **(B)** Relative abundance histogram of the top 10 genera in each BMI group. **(C)** LEfSe results showing linear discriminant analysis (LDA) scores (LDA > 2.0) and corresponding heatmap of significantly different genera across groups. Ob, *n* = 38; Ow, *n* = 91; Nor, *n* = 85. Group differences were evaluated using the Kruskal–Wallis test with Benjamini–Hochberg false discovery rate correction. Colored diamonds indicate intergroup statistical comparisons (adjusted *p*-values).

Microbial composition analysis identified 330 genera spanning 16 phyla across all samples. The top 10 phyla by average relative abundance are presented in Figure 2A, with Bacillota demonstrating the highest dominant phyla (Ob: 53.7%; Ow: 52.2%; Nor: 58.5%) and no significant intergroup differences (*p* > 0.05). Pseudomonadota, Bacteroidota, and Actinomycetota were similarly dominant across cohorts. At the genus level, *Bacteroides*, *Escherichia-Shigella*, and *Faecalibacterium* exhibited the highest relative abundances. Comparative analysis revealed *Bacteroides* and *Faecalibacterium* were enriched in the Nor group, whereas *Escherichia-Shigella* showed an inverse distribution pattern (Figure 2B). LefSe analysis (LDA score > 2, *p* < 0.05) combined with relative abundance heatmap (Figure 2C) identified distinct microbial markers across groups. The Ob group exhibited significant enrichment of *Escherichia-Shigella*, *Collinsella*, *Lactobacillus*, and *Parabacteroides*, with *Escherichia-Shigella* and *Lactobacillus* showing higher abundance in Ob compared to both Ow and Nor groups. *Parabacteroides* abundance in Ob was significantly elevated versus Nor but did not differ from Ow. Conversely, the Ow group demonstrated enrichment of *Ruminococcus gnavus group*, *Morganella*, *Clostridium innocuum group*, and *Hungatella*. Thirteen genera including *Faecalibacterium*, *Enterobacter*, and *Agathobacter* were preferentially enriched in Nor, with most showing significantly higher abundance than both Ob and Ow groups (except *Erysipelotrichaceae UCG-003*). These findings indicate that genus-level disparities primarily distinguish Nor from both Ob and Ow groups.

### 3.4 Correlation analysis between gut microbial and clinical indicators

**Figure 3:**
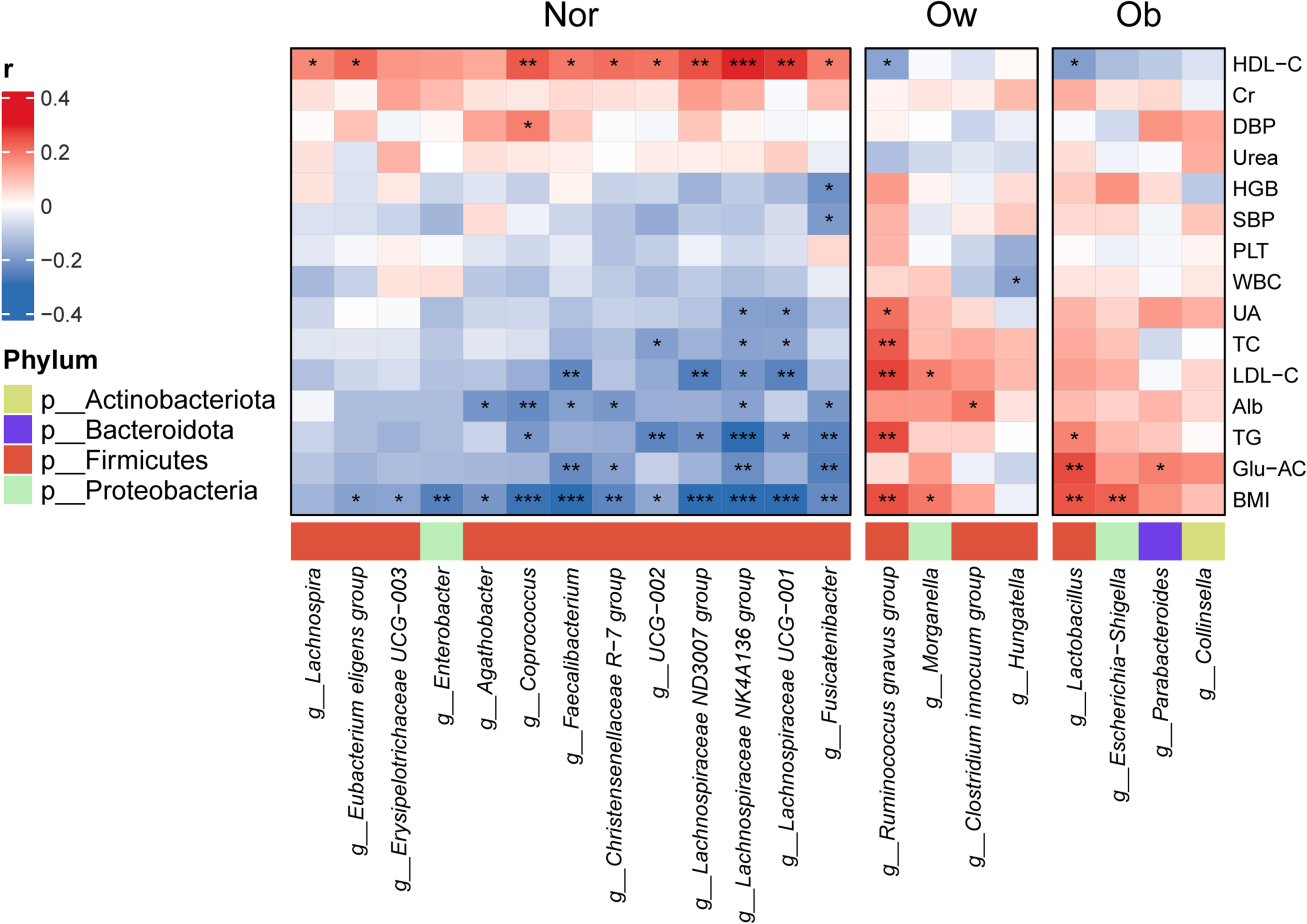
Heatmap showing spearman correlation coefficients between significantly altered genera and clinical parameters (BMI, TG, LDL-C, HDL-C, Glu-AC, Alb, UA, etc.). Red indicates positive correlation and blue indicates negative correlation. Statistical significance: * p < 0.05, ** p < 0.01, *** p < 0.001 (FDR-adjusted).

Spearman correlation analysis between LEfSe-identified differential bacterial genera and clinical indicators revealed BMI-associated microbial signatures across groups (Figure 3). In the Nor cohort, *Coprococcus*, *Faecalibacterium*, *Lachnospiraceae UCG-001*, *Lachnospiraceae NK4A136 group*, and *Lachnospiraceae ND3007 group* exhibited significant inverse correlations with BMI (*p* < 0.001). Conversely, the Ob group demonstrated positive correlations for *Lactobacillus* and *Escherichia-Shigella*, while Ow individuals showed similar positive associations with *Ruminococcus gnavus group* and *Morganella* abundance. Significant microbiota-clinical correlations were observed across BMI groups (Figure 3). In the Nor cohort, four genera, including *Faecalibacterium* and *Fusicatenibacter*, exhibited negative associations with Glu-AC, while six genera, including *Lachnospiraceae NK4A136 group* and *Lachnospiraceae UCG-001*, showed inverse correlations with TG. Six genera including *Coprococcus* were negatively associated with Alb, and four genera, including *Faecalibacterium* and *Lachnospiraceae UCG-001*, demonstrated negative correlations with LDL-C. Ten genera, including *Lachnospiraceae NK4A136 group* and *Coprococcus*, were positively correlated with HDL-C. Conversely, the Ow and Ob cohorts displayed reversed patterns. In the Ow group, the *Ruminococcus gnavus group* showed positive correlations with TG, LDL-C, TC, and UA but negative with HDL-C, while *Morganella* was positively associated with LDL-C. *Clostridium innocuum group* tracked positively with Alb, and *Hungatella* inversely with WBC. In the Ob group, *Lactobacillus* was positively correlated with Glu-AC and TG yet negatively with HDL-C, paralleled by *Parabacteroides*’s positive association with Glu-AC.

### 3.5 Clinical indicators improve diagnostic performance of bacterial markers

**Figure 4:**
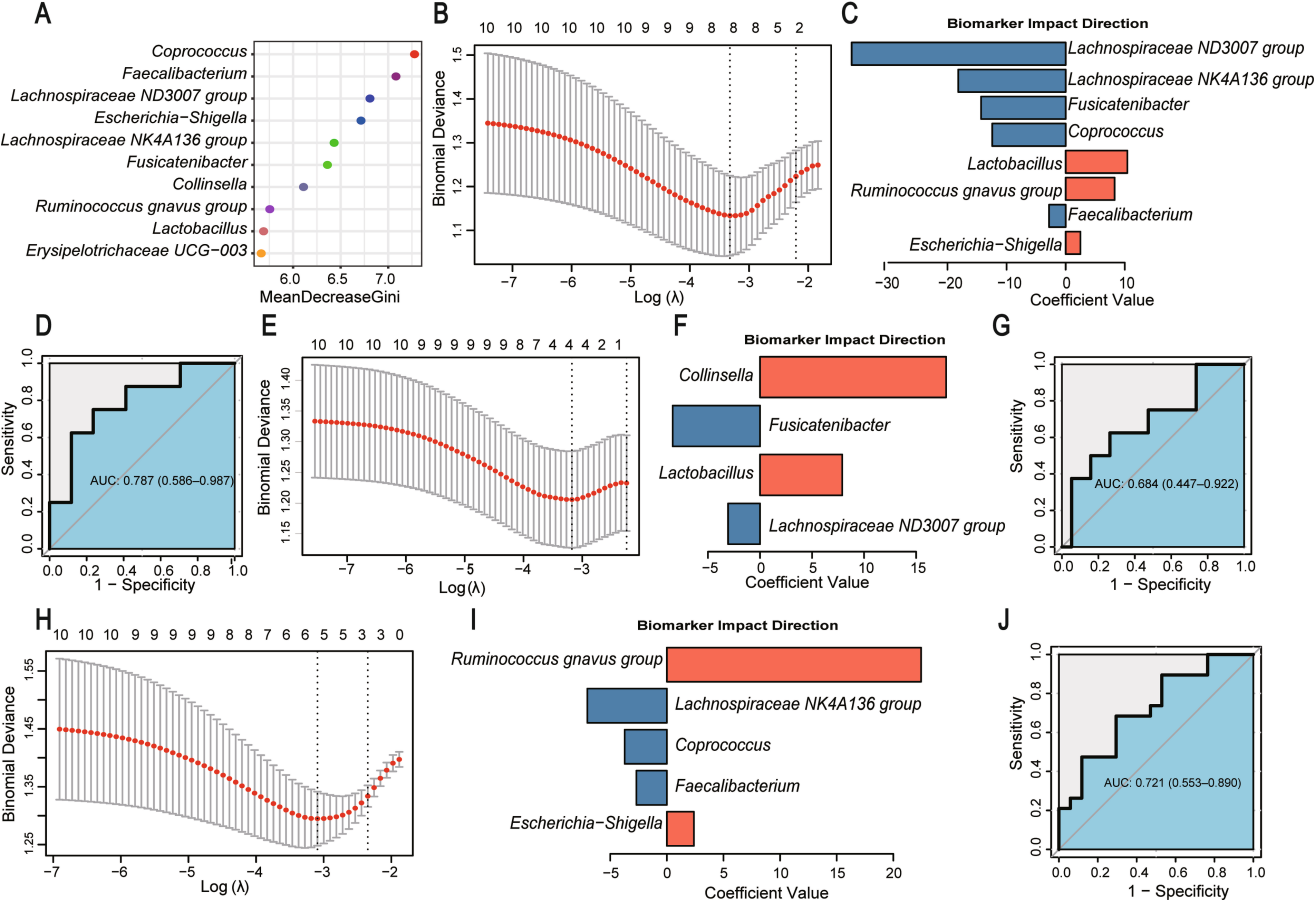
Identification of gut microbial biomarkers distinguishing BMI groups. **(A)** Mean decrease Gini (MDG) values from random forest ranking the top 10 discriminatory microbial genera. **(B)** LASSO regression model for Nor vs. Ob classification. **(C)** Coefficients of selected composite microbial markers (blue: negatively associated with Ob; orange: positively associated). **(D)** ROC curve for Nor vs. Ob discrimination. **(E)** LASSO model for Ob vs. Ow classification. **(F)** Coefficients of microbial markers distinguishing Ob from Ow. **(G)** ROC curve for Ob vs. Ow classification. **(H–J)** LASSO model, coefficients, and ROC curve for Nor vs. Ow classification. AUC indicates area under the ROC curve; 95% confidence intervals are shown in brackets. All models were trained using stratified 10-fold cross-validation, and *p*-values were FDR-corrected, ROC curves and AUC values were derived from Firth-corrected models.

LEfSe analysis identified 21 differential microbial markers (Figure 2C), from which potential biomarkers for distinguishing Ow and Ob were selected using random forest analysis (Figure 4A). Subsequent LASSO regression refined the discriminatory microbial markers in the gut microbiome. Based on the top 10 species identified by random forest, we identified eight intestinal microorganisms that effectively differentiated the Nor and Ob groups, including *Lachnospiraceae ND3007 group*, *Lachnospiraceae NK4A136 group*, *Fusicatenibacter*, *Coprococcus*, *Lactobacillus*, *Ruminococcus gnavus group*, *Escherichia-Shigella*, and *Faecalibacterium*. The composite microbial marker demonstrated diagnostic potential with an AUC of 0.787 (Figures 4B–D). Furthermore, four markers including *Collinsella*, *Fusicatenibacter*, *Lactobacillus*, and *Lachnospiraceae ND3007 group* were effectively differentiated Ob from Ow, with an AUC value of 0.684 (Figures 4E–G). Similarly, five microorganisms including *Ruminococcus gnavus group*, *Lachnospiraceae NK4A136 group*, *Coprococcus*, *Faecalibacterium*, *Escherichia−Shigella*, effectively distinguished Nor from Ow individuals, achieving an AUC of 0.721 (Figures 4H–J).

**Figure 5:**
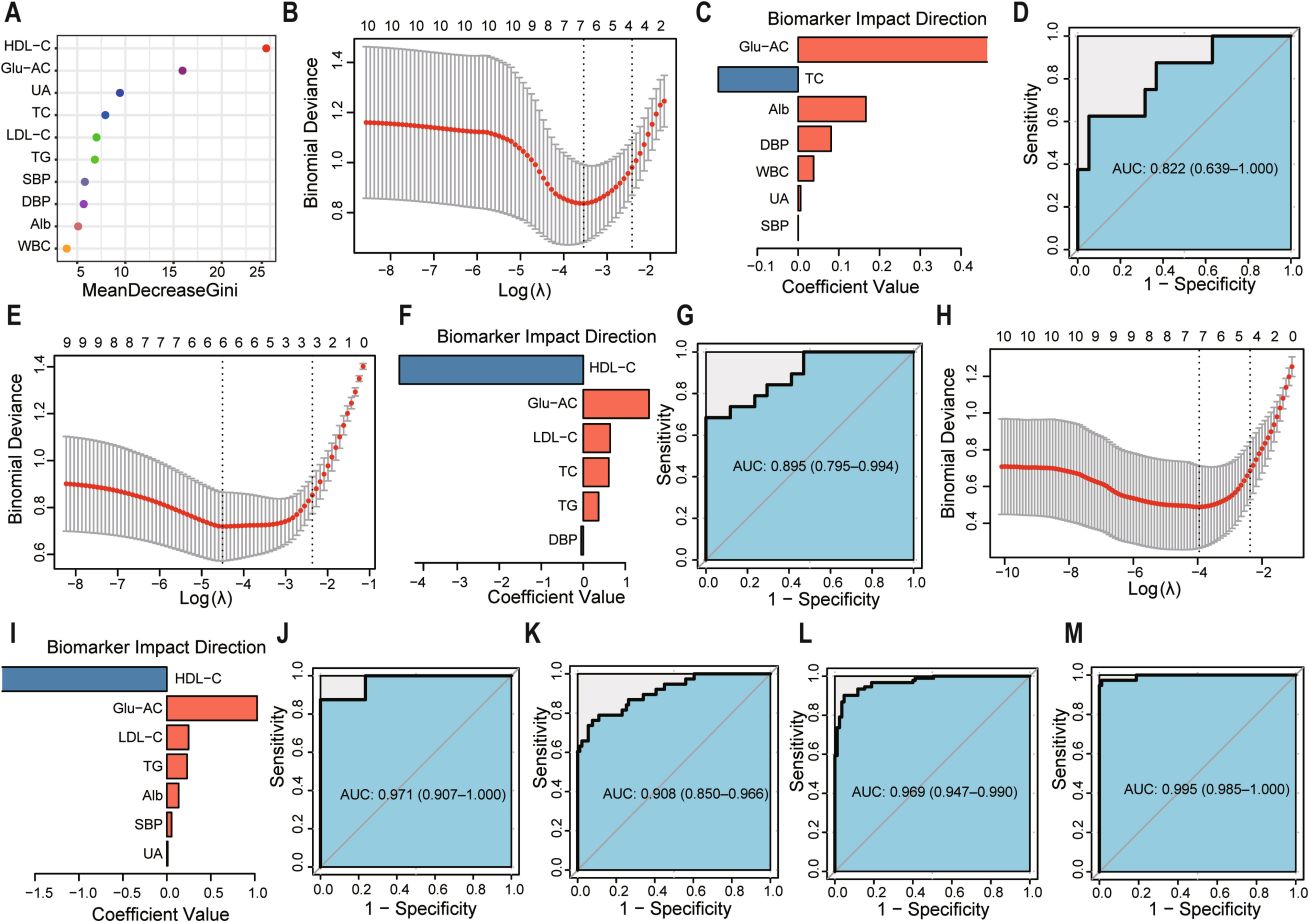
Enhancement of BMI-group discrimination through clinical indicators alone and combined with microbial markers. **(A)** Mean decrease Gini (MDG) values ranking the top 10 discriminative clinical indicators (excluding BMI) in random forest analysis. **(B)** LASSO regression model for Ob vs. Ow classification. **(C)** Coefficients of selected clinical predictors. **(D)** ROC curve for Ob vs. Ow classification. **(E–G)** LASSO model, coefficients, and ROC curve for Nor vs. Ob classification. **(H–J)** LASSO model, coefficients, and ROC curve for Nor vs. Ow classification. **(K–M)** ROC curves for combined microbial–clinical models for Ob vs. Ow **(K)**, Nor vs. Ow **(L)**, and Nor vs. Ob **(M)**. All AUC values include 95% confidence intervals. All analyses were performed using stratified 10-fold cross-validation, and *p*-values were FDR-corrected, ROC curves and AUC values were derived from Firth-corrected models.

In addition, random forest analysis identified 10 key clinical indicators (excluding BMI) for distinguishing Ow and Ob (Figure 5A). LASSO regression revealed seven indicators (Glu-AC, TC, Alb, DBP, WBC, UA, SBP) that effectively differentiated Ob from Ow, achieving an AUC of 0.822 (Figures 5B–D). Similarly, six indicators (HDL-C, Glu-AC, LDL-C, TC, TG, DBP) distinguished Nor from Ob with perfect discrimination (AUC = 0.895; Figures 5E–G), while seven indicators (HDL-C, Glu-AC, LDL-C, TG, Alb, SBP, UA) differentiated Nor from Ow (AUC = 0.971; Figures 5H–J). The diagnostic performance was further enhanced through combined analysis. Integrating key clinical indicators with bacterial biomarkers improved discrimination between Ob and Ow (AUC increased from 0.684 to 0.908; Figure 5K). Similarly, combined models for Nor vs. Ow and Nor vs. Ob achieved AUC values of 0.969 and 0.995, respectively (Figures 5L,M). These findings demonstrate the synergistic potential of clinical indicators and microbial biomarkers for early obesity detection.

## 4 Discussion

Over the past decade, the gut microbiota has been increasingly recognized as a key contributor to obesity pathogenesis. Substantial evidence demonstrates that obese individuals exhibit significant alterations in microbial composition, functional capacity, and metabolic output, particularly characterized by reduced butyrate-producing bacteria, elevated pro-inflammatory taxa, and impaired SCFAs biosynthesis (Bouter et al., 2017; Sun et al., 2018; Aron-Wisnewsky et al., 2021). However, as research cohorts expand and analytical techniques advance, the conventional definition of an “obesity-associated microbiome” requires critical reevaluation (Magne et al., 2020). Critically, most prior studies have treated obesity as a single state (e.g., obesity vs. normal) or focused exclusively on extreme BMI groups, overlooking the transitional “overweight” phase. From a public health perspective, this intermediate stage represents both a precursor to obesity and the most reversible window for microbial ecological interventions. Our study provides novel insights into the gut microbiota’s role in obesity by focusing on the often-overlooked overweight population and by constructing a diagnostic framework that deliberately excludes BMI.

Our results demonstrate that Ob exhibits a significantly lower Shannon index compared to Nor, reflecting reduced *α*-diversity of gut microbiota—a finding consistent with prior research (Hu et al., 2022). It is noteworthy that the absolute values of the Shannon index in our cohort (primarily ranging between 2 and 3.7 for the Nor group) appear lower than some published benchmarks. This discrepancy may be attributed to methodological variations (e.g., DNA extraction kits, 16S rRNA gene region targeted [V3-V4], sequencing platform, and bioinformatic pipelines), population-specific factors (e.g., genetic background, geography, and diet of our Chinese cohort), or a combination thereof. While cross-study comparisons of absolute diversity values should be interpreted with caution, the significant relative difference observed between the Ob and Nor groups within our standardized analytical framework remains valid and informative. Notably, no significant α-diversity difference was observed between the Ow and Ob groups. *β*-diversity analysis revealed a gradient distribution of microbial communities across BMI categories, with Ob and Nor groups forming distinct clusters and Ow positioned intermediately, suggesting BMI as a key driver of microbiota restructuring (Wang et al., 2025).

At the genus level, *Coprococcus*, *Faecalibacterium*, *Lachnospiraceae UCG-001*, and *Lachnospiraceae NK4A136 group* were significantly enriched in the Nor group. Notably, *Faecalibacterium*-a genus consistently linked to metabolic health-exhibits reduced abundance in obese individuals, as demonstrated by prior studies (Maioli et al., 2021; Islam et al., 2022). The *Lachnospiraceae NK4A136 group* and *Lachnospiraceae UCG-001* exhibited a positive correlation with SCFA production capacity, a metabolic pathway critically linked to enhanced energy metabolism and attenuated inflammatory responses (Ma et al., 2020). Furthermore, our findings corroborate prior evidence indicating an inverse association between the *Lachnospiraceae NK4A136 group* and obesity (Companys et al., 2021). Notably, while certain studies report increased *Coprococcus* abundance in obese individuals, significant functional heterogeneity exists within this genus. Multiple independent cohort and intervention studies demonstrate that specific species (e.g., *C. eutactus*) correlate with enhanced insulin sensitivity, reduced BMI, and possess butyrate-producing and anti-inflammatory capabilities (Yang et al., 2023; González et al., 2025). Thus, the *Coprococcus* enrichment observed in our normal-weight group likely represents a “beneficial subpopulation,” with its depletion potentially indicating loss of functionally protective microbiota in obesity. However, the *Ruminococcus gnavus group*, *Morganella*, and *Clostridium innocuum group* were significantly enriched in the Ow cohort. Notably, both the *Ruminococcus gnavus group* and *Morganella* are associated with pro-obesity effects (Grahnemo et al., 2022; Sun et al., 2023), whereas the *Clostridium innocuum group*-a recognized probiotic-exhibits intestinal regulatory and potential anti-obesity properties (Bian et al., 2022; Mei et al., 2022). These findings suggest that the gut microbiota in the Ow stage may represent a transitional equilibrium, characterized by the concurrent expansion of pro-inflammatory taxa and compensatory proliferation of protective flora. The *Escherichia-Shigella*, *Collinsella*, and *Parabacteroides* genera were significantly enriched in the Ob group. *Escherichia-Shigella* exhibited obesogenic and pro-inflammatory properties in the gut of obese individuals, showing significant positive correlations with obesity markers including BMI (Hong et al., 2024; Gao et al., 2025). Similarly, *Collinsella* demonstrated consistent positive associations with obesity-related parameters such as TG, TC, and BMI (Gallardo-Becerra et al., 2020; Wang et al., 2020). While *Parabacteroides* is generally regarded as a potentially beneficial genus, certain studies have reported its positive association with BMI (Chiu et al., 2014; Guan et al., 2021). Notably, *P. merdae* and *P. distasonis* demonstrate significant enrichment in individuals with hypertension and diabetes, with *Parabacteroides* exhibiting particularly high abundance in obese patients with type 2 diabetes. These findings suggest that specific *Parabacteroides* species may contribute to metabolic dysregulation (Yan et al., 2017; Hasain et al., 2020; Yang et al., 2021). The *Parabacteroides* genus demonstrates significantly higher abundance in individuals with inflammatory bowel disease or elevated inflammatory indices, suggesting a potential association between *Parabacteroides* proliferation and systemic inflammation levels (Aranaz et al., 2021; Iliodromiti et al., 2023). This observation aligns with the elevated WBC counts consistently reported in obese populations, which further corroborates systemic inflammatory responses. Notably, our findings reveal increased *Parabacteroides* abundance in obese individuals, potentially indicating the expansion of specific *Parabacteroides* subspecies linked to metabolic dysregulation and pro-inflammatory alterations in the gut microbiota. Of particular note is that the abundance of *Lactobacillus* was significantly increased in the obese group, a result that seems to contradict the “beneficial” image of this genus that is often widely used in probiotic preparations. However, in fact, the *Lactobacillus* genus is highly heterogeneous, and different species and even different strains of the same species have significant differences in their effects on host metabolism. For example, *Lactobacillus acidophilus* has been shown to be associated with weight gain, while *Lactobacillus plantarum* has been shown to improve obesity (Million et al., 2012). Furthermore, a study found that the number of *Lactobacillus* species in obese patients was significantly higher than lean controls, a finding consistent with our results (Armougom et al., 2009). However, the limitations of 16S rRNA sequencing preclude precise species-level differentiation. We therefore hypothesize that the enriched *Lactobacillus* population in obesity may represent an obesity-promoting subpopulation or strains opportunistically amplified under metabolic stress. These observations underscore the need to avoid oversimplified classifications of *Lactobacillus* as uniformly “beneficial” or “harmful,” instead advocating for functional subpopulation characterization through future strain-level analyses.

The Spearman correlation network we constructed revealed the multi-layer associations between differential bacterial genera and clinical metabolic indicators. In the Nor group, butyrate-producing bacteria including *Faecalibacterium*, *Coprococcus*, *Lachnospiraceae ND3007 group*, and *Lachnospiraceae NK4A136 group* were significantly enriched. These genera exhibited negative correlations with BMI, TG, and LDL-C, while demonstrating positive associations with HDL-C. These observations align with established evidence that elevated butyrate levels correlate with reduced adiposity and inverse associations with obesity markers and blood glucose levels (Amiri et al., 2023; Zhang et al., 2024). These findings are consistent with our results.

However, the *Ruminococcus gnavus group* in the Ow cohort exhibited inverse correlations with obesity-related metabolic markers (BMI, TC, and LDL-C) compared to the Nor group. This bacterial group has been widely recognized as a potential biomarker for multiple pathologies, including obesity, depression, and inflammatory bowel disease (Henke et al., 2019; Juge, 2023; He et al., 2025). Its elevated abundance in obese individuals aligns with existing evidence linking *R. gnavus* to adverse metabolic phenotypes, such as increased adiposity, elevated waist circumference, hypertriglyceridemia, and reduced HDL-C levels (Grahnemo et al., 2022). Notably, *Hungatella* exhibits a negative correlation with WBC counts, implying its potential protective role in modulating host inflammatory responses. This observation aligns with existing evidence demonstrating *Hungatella*’s capacity to enhance intestinal barrier function and immune regulation through SCFA production, which may suppress low-grade inflammation (Xing et al., 2025). While the exact mechanisms are not yet fully understood, these findings indicate that *Hungatella* may engage in compensatory upregulation of anti-inflammatory pathways in response to overweight conditions. This hypothesis merits additional research. Notably, the *Escherichia−Shigella* genus was significantly enriched in the Ob group and exhibited a positive correlation with BMI-a finding consistent with prior reports (Hong et al., 2024; Gao et al., 2025). While most studies associate *Lactobacillus* with metabolic benefits, our data revealed its positive correlation with Glu-AC and TG levels, aligning with limited but corroborative evidence (Li et al., 2022; Crudele et al., 2023; Feng et al., 2023). This discrepancy suggests strain- or host-specific metabolic effects of *Lactobacillus*. Conversely, *Parabacteroides* showed exclusive positive correlation with Glu-AC, potentially reflecting its dietary adaptation to high-fat/high-sugar conditions (de Wouters d'Oplinter et al., 2021).

Utilizing machine learning models, we successfully identified bacterial genera capable of distinguishing BMI stratification, with model AUC values ranging from 0.721 to 0.787. Notably, integrating these microbial markers with conventional clinical metabolic indicators (e.g., TG, HDL-C, UA) substantially enhanced diagnostic performance. It is important to highlight that, given that BMI has been utilized as the primary grouping variable in this research, this approach serves to mitigate the potential for pseudo-correlations arising from the interaction of cross-variables within the model, on the one hand, BMI alone often inadequately classifies obesity-related diseases at the individual level, as individuals with similar BMI values frequently exhibit divergent metabolic phenotypes (Coral et al., 2025). Therefore, we deliberately excluded BMI from the diagnostic model during the modeling process to test whether other metabolic indicators have independent stratification capabilities. The results demonstrated that key metabolic indicators (including TG, HDL-C, and UA) significantly enhanced the model’s discriminative performance independent of BMI, confirming their intrinsic predictive value for obesity risk stratification. Notably, when integrated with microbial markers, the combined model exhibited a substantial increase in AUC values, indicating that this “microbiome-metabolic” framework maintains robust diagnostic potential without BMI data. These findings highlight the clinical translatability of this integrated approach for non-invasive metabolic risk assessment.

While this study provides novel insights into structural variations of gut microbiota across different BMI categories, several limitations should be acknowledged. First, the cross-sectional design precludes causal inference between microbial alterations and metabolic indicators. While we propose that the microbial signatures identified in the overweight group hold potential for early risk stratification, this cross-sectional snapshot cannot demonstrate that these signatures actually predict future progression to obesity. Future studies incorporating longitudinal follow-up of overweight individuals are essential to validate the predictive power of these microbial markers for true early risk stratification. Such studies would track whether individuals with the ‘dysbiotic’ overweight profile we identified are indeed more likely to develop obesity and associated metabolic disorders over time. Second, the use of 16S rRNA sequencing limits taxonomic resolution and does not allow species- or strain-level differentiation. Thus, the observed associations—such as those involving Lactobacillus—may reflect underlying strain variability that cannot be resolved with 16S data alone. Future work using metagenomic approaches will be required to validate these findings and characterize functional differences more accurately. Third, key lifestyle factors (e.g., diet, physical activity, and sleep patterns) were not systematically incorporated into the analysis. These variables not only influence gut microbiota composition but may also confound the observed associations between BMI and microbial profiles. Future studies should employ comprehensive dietary frequency surveys and energy intake assessments, coupled with standardized exercise and sleep monitoring, to mitigate confounding effects and strengthen the model’s explanatory power. Additionally, the study’s single-center design and geographically restricted sampling may limit the generalizability of the findings. Given that gut microbiota composition is influenced by multifactorial determinants (e.g., genetics, diet, and environmental exposures), future multi-center studies involving diverse ethnic populations are warranted to validate and enhance the external validity of these conclusions.

## 5 Conclusion

This study revealed BMI-related alterations in gut microbiota composition and diversity, and demonstrated that integrating microbial features with clinical indicators enhances obesity stratification beyond BMI alone. These findings provide valuable reference evidence for the early stratification of obesity risk, suggesting that microbiota-informed assessment may complement traditional anthropometric measures. To advance toward practical application, future studies should include longitudinal cohorts, expanded sample sizes, and higher-resolution microbial profiling to clarify causal relationships and to determine whether these microbial signatures can reliably support early risk identification or guide individualized intervention strategies.

## Data Availability

The 16S rRNA gene sequencing data generated in this study have been deposited in the NCBI BioProject database under accession number PRJNA1394711.
